# Nitrogen immobilization may reduce invasibility of nutrient enriched plant community invaded by *Phragmites australis*

**DOI:** 10.1038/s41598-020-58523-4

**Published:** 2020-01-31

**Authors:** Md Nazim Uddin, Randall William Robinson, Takashi Asaeda

**Affiliations:** 1 Institute for Sustainable Industries & Liveable Cities, College of Engineering & Science, Victoria University, Melbourne, Victoria 8001 Australia; 2 Department of Environmental Science & Technology, Saitama University, 255 Shimo-Okubo, Sakura-Ku, Saitama 338-8570 Japan; 3 Institute for Studies of the Global Environment, Sophia University, 7-1 Kioicho, Chiyoda, Tokyo 102-8554 Japan

**Keywords:** Wetlands ecology, Restoration ecology

## Abstract

Nutrient enrichment, particularly nitrogen, is an important determinant of plant community productivity, diversity and invasibility in a wetland ecosystem. It may contribute to increasing colonization and dominance of invasive species, such as *Phragmites australis*, especially during wetland restoration. Providing native species a competitive advantage over invasive species, manipulating soil nutrients (nitrogen) may be an effective strategy to control the invasive species and that management tool is essential to restore the degraded ecosystems. Therefore, we examined competition between *Phragmites australis* and *Melaleuca ericifolia* in a greenhouse setting with activated carbon (AC) treatments, followed by cutting of *Phragmites* shoots in nutrient-rich soils. Additionally, we evaluated the effect of AC on plant-free microcosms in the laboratory, to differentiate direct effects of AC on soil microbial functions from indirect effects. Overall, the objective was to test whether lowering nitrogen might be an effective approach for reducing *Phragmites* invasion in the wetland. The AC reduced *Phragmites* total biomass more significantly in repeated cut regime (57%) of *Phragmites* shoots compared to uncut regime (39%). Conversely, it increased *Melaleuca* total biomass by 41% and 68% in uncut and repeated cut regimes, respectively. Additionally, AC decreased more total nitrogen in above-ground biomass (41 to 55%) and non-structural carbohydrate in rhizome (21 to 65%) of *Phragmites*, and less total nitrogen reduction in above-ground biomass (25 to 24%) of *Melaleuca* in repeated cut compared to uncut regime. The significant negative correlation between *Phragmites* and *Melaleuca* total biomass was observed, and noticed that *Phragmites* acquired less biomass comparatively than *Melaleuca* in AC-untreated versus AC-treated pots across the cutting frequency. AC also caused significant changes to microbial community functions across *Phragmites* populations, namely nitrogen mineralization, nitrification, nitrogen microbial biomass and dehydrogenase activity (*P* ≤ 0.05) that may potentially explain changes in plant growth competition between *Phragmites* and *Melaleuca*. The overall effects on plant growth, however, may be partially microbially mediated, which was demonstrated through soil microbial functions. Results support the idea that reducing community vulnerability to invasion through nutrient (nitrogen) manipulations by AC with reducing biomass of invasive species may provide an effective strategy for invasive species management and ecosystem restoration.

## Introduction

There are numerous global environmental issues ranging from climate change, global warming, pollution, biological invasion to loss of biodiversity and many more^1^. Amongst these, biological invasion has been considered the most persistent issue to cause biodiversity loss and species extinction placing negative impacts on natural communities and ecological characteristics^2,3^. In parallel, nutrient enrichment is augmenting the invasion process, and is considered a communal obstacle in implementing plant community restoration initiatives^4^. Human-induced fertilization due to industrialization^5^, atmospheric nitrogen deposition through air pollution^6^, and invasion by N-fixing shrubs^7^ can influence the increase in soil nutrients, in particular N availability. As a result, the nutrient enrichment in soil can benefit fast-growing invasive plant species, that outperform the native slow-growing plant species and invade into native ecosystems^8^. Wetlands, including brackish marshes, represent one of the most productive natural habitats^9^ supporting various biological communities and biogeochemical functions. These provide heterogeneous nutrient conditions and diverse habitats, which can play as one of the key drivers to global nutrient cycling^9,10^. Additionally, topographic features of wetlands make these ecosystems vulnerable to human-induced disturbances^11^, thereby, facilitating invasion of non-native plants, and even some native plants play dominating role into the ecosystem.

Invasion of *Phragmites australis* (hereafter *Phragmites*) possess the great threat amongst aggressive and widely studied invasive plants in wetlands^12,13^. It significantly modifies and often destroys ecosystem structure and functions^14–17^. Physiological characteristics of *Phragmites* including high rates of reproduction, well-developed aerenchyma and rhizosphere facilitate colonization, and dense canopies related to their high productivity can inhibit germination and growth of neighbour plant species^18–20^. The tissue chemistry of *Phragmites* and its extensive production alters nutrient pools^21^ and availability in soils^16,18^. Together with its negative effects on ecology, *Phragmites* can impose considerable financial loss^22^. For example, invasive species management costs about $25 billion/year in the USA^23^. Globally, there are numerous wetland restoration projects related to *Phragmites* invasion representing significant cost to the USA, Canada and Australia^24–26^. Though invasiveness is dependent upon genotypes, for example, a Eurasian genetic lineage is considered as an invader in North America^27^ and *Phragmites* has been considered a cryptic invader creating management problems for scientists and land managers^28,29^. The invasive plant management project is generally lacking scientific information regarding invasion mechanism of the targeted species. For example, *Phragmites* is considered a cryptic invader, it may lack specific information related to invasion mechanisms, though it depneds on its context^30^. Current methods to control *Phragmites* invasions are aimed at eradication of existing populations, which are effective only in short term^31^. The recent study raises the question about the efficacy of managing large populations invaded by *Phragmites* through the method^22^. Generally, *Phragmites* populations can be treated using herbicide applications in combination with cutting, burning and covering with plastic^32^. *Phragmites* populations may quickly re-sprout from rhizome and seed bank reserves after the treatments^33^, indicating it requires long-term monitoring and re-treatment until native vegetation can be restored. Some recent studies showed seeds compared to vegetative propagules may play an important role to reproduce the *Phragmites* colonization^27^. Thus, repeating control measures indefinitely to address recurring *Phragmites* invasions is not only expensive, and time consuming, but also interferes with native plant community establishment^34^.

There are many studies supporting the assertion that nutrient enrichment favours invasiveness and long-term persistence of the invader in wetland native plant communities making the community more susceptible to be invasible^35–37^. The approach to ecosystem management like *Phragmites* invaded ecosystems, focus on simply eradicating *Phragmites* stands rather than nutrient management^38,39^ and restoring native plant communities^32^. Again, very few studies have been conducted systematically on community invasibility to compare the effects of disturbance regimes^40^ and how management approaches may work effectively on promoting re-establishment of neighbouring plant species^4^. Therefore, we hypothesized that creating conditions through nutrient (nitrogen) manipulation in soil followed by frequent cuttings of *Phragmites* shoots may favour associated plant species *Melaleuca ericifolia* (hereafter *Melaleuca*) over *Phragmites* that would be a more operative approach to reduce invasions by *Phragmites*. The *Melaleuca* has been selected as an alternative plant for wetland restoration because distribution and abundance of this indigenous plant has reduced sharply due to high salinity, nutrient enrichment, flooding and invasion along the entire coastal zone of Australia, especially southern-eastern Australia^39,41,42^. Thus, the rehabilitation of those wetlands is the priority of the natural resource management agencies (government and private) in Australia^43,44^. This hypothesis is associated with the resource–ratio hypothesis, that explains plant community composition influenced by relative abundance of limiting resources in soil^45,46^. Thus, the resource (nutrient) manipulation limits the availability to the target dominating plant species, but it may benefit the desired native species^47^. Our previous studies support the idea that nutrient enrichment, specifically high nitrogen (N) availability, can facilitate *Phragmites* invasion into wetlands and thereby, suppress the associated plant species *Melaleuca*^39^. In addition to N enrichment, increased phosphorus may play an important role to displace the slow-growing native plants by fast-growing invasive plants in many ecosystems including wetland ecosystems^48,49^. But, in particular, N enrichment in the wetland is the key driver to play community invasibility because a large part of N is being deposited in wetlands through surface runoff and ground water^50^. Thus, *Phragmites* is highly responsive to N availability and it attains a competitive advantage under these conditions^39^ similar to plant species *Rumex confertus*^51^.

The key matter in restoration with nutrient-enriched ecosystems is to deal with the question of how the slower growing native species may benefit in competition over fast-growing invasive species^52,53^. Till now, there is no conclusive assertion about the successful mechanisms of invasive species and the susceptibility of plant communities to invasion^54,55^. Therefore, documentation of species-specific invasion mechanism, its effects and control into invaded plant communities is more reasonable and methodical than on general theories for all invasive species^54,56,57^. In addition, relatively very little research has focused on the effects of control measures of the invasive species, for example, *Phragmites* on the rhizome and rhizosphere soil that influence plant productivity, and likely contribute to competitive advantages over native plant species^58^. Therefore, we questioned whether depletion of rhizome reserves via shoot cutting and changes in rhizosphere soil characteristics through soil nitrogen immobilization via activated carbon (AC) amendment might be the key to control of *Phragmites* in its invaded communities. Activated carbon addition to soil generally reduces nitrogen availability to plants^59^. The general assumption is that AC reduces inorganic nitrogen content in soil such as NO_3_ and NH_4_^60^ which are mostly available for plant growth and they may limit growth and productivity of plant^61^. The underlying mechanism is that addition of AC leads to nitrogen immobilization through greater microbial N uptake^62^, but it depends on soil characteristics^63^. The general understanding is that limiting N availability to plant through immobilization may reduce the productivity of the invasive plant, and thereby, increasing the growth of native ones, which also depends on the response of the plant to N availability in soil^64^.

Therefore, we conducted greenhouse experiments to test whether management approaches in nutrient enriched condition may increase the competitive ability of *Melaleuca* over *Phragmites* into a wetland plant community. Additionally, a laboratory soil incubation experiment was conducted to test whether AC-induced changes to soil microbial functions via nitrogen immobilization could be helpful to explain the changes in plant growth in the above experiments. Our precise objectives were to (i) study the interactive effects of AC amendment in growth substrate and repeated shoot cutting of *Phragmites* on the competitional growth between *Phragmites* and *Melaleuca*, and (ii) assess whether responses of plant productivity and resources (carbohydrate storage, nitrogen concentration) to AC addition and subsequent cutting of *Phragmites* shoots were mediated by N immobilization via microbial interactions. More specifically, the experiments were conducted to test whether AC addition followed by repeated cutting of *Phragmites* shoots either individually or combinedly could facilitate *Melaleuca* success given sufficient competition over *Phragmites*.

## Materials and Methods

### Study species

*Phragmites australis* is a cosmopolitan and successional species that is distributed all over the world (except Antarctica), and still it remains unclear about its originality in many regions of the earth^21^. It has been considered as a weedy and invasive population in North America, Australia, and Madagascar^65–67^. *Phragmites* is widely-dominated across most parts of temperate regions of Australia^65^. It forms mostly dense monospecific stands in fresh and coastal wetlands, freshwater swamps and lakes, along rivers, and even in irrigation canals^68^. On the other hand, *Melaleuca* is a native shrub or small tree with a maximum height 8 m, is distributed in coastal and near coastal freshwater and brackish-water wetlands across southeastern Australia^44^. Most species of the genus *Melaleuca* are reliant on seed alone for reproduction; however, *Melaleuca* can form clonal stands through the production of ramets^69^. These two species naturally coexist in most of the wetlands in south-eastern Australia^70,71^.

### Greenhouse studies

#### Collection of plant materials

Methods for the greenhouse experiments were followed according to our previous works^39,42^ with modifications in treatments. Briefly, in September 2012, we collected rhizomes of *Phragmites* from Cherry Lake (37°51′30″S, 144°50′5″E), that is a coastal wetland in Altona, Melbourne, Victoria, Australia^72^. Cherry Lake was a low land and seasonally inundated marshy area before European settlement, but following urbanization, it was altered and drained to manage the wetland. During European settlement, *Phragmites* occupied a small portion of the wetland, but gradually, it has been expanding vigorously and making almost monospecific stands that changes the floristic composition^16^. Considered a less-disturbed and low-nutrient wetland^39^, the site was selected to source plant materials. We used the live rhizome for the experiment consisting of only one active node. We purchased 6-month old *Melaleuca* seedlings from a commercial source, grown from locally collected seeds in potting mix soil^42^.

#### Soil preparation

We used circular 7 L plastic pots (25 × 22 × 17-cm) containing 4 L substrate. We prepared the substrate with 1:7 ratio of river sand and potting mix respectively, including nutrient addition treatment (3 g/L) nutrient level. The composition of the potting mix was organic materials, living organisms, minerals and nutrients (see ‘availability of materials and data’ for more information). The used nutrient was the mixed pelletized fertilizer with a ratio of N-P-K: 16-8-9 and it was used to enrich nutrient level. In addition, a 50 mL of soil from mixed population of *Phragmites* and *Melaleuca* in Edithvale wetland (described below in details) was added to the substrate for introduction of microbes. This soil was also used for a later field soil incubation experiment. We mixed soil (river sand & potting mix) with inoculum and nutrients appropriately in isolated pots by hand, placing the mixture into targeted plastic pots, then used for transplantation of the excised rhizomes of *Phragmites*.

#### Experimental design and measurement

To examine the interactive effects of AC amendment in soil substrate and repeated shoot cutting of *Phragmites* on competition between experimental plants in nutrient enriched condition, we initially manipulated the substrate pots with AC (activated charcoal powder made from coconut shell) [with (70 g/L substrate) and without AC (0 g/L)] and nutrient (3 g/L). The manipulated pots were then subjected to nutrient addition at a rate of 3 g/L in the tilled topsoil monthly. Plants were arranged in treatment pots as *Phragmite*s alone, *Melaleuca* alone, and *Melaleuca* with *Phragmite*s with five replicates. The management strategies of the experimental pots were: (a) *Phragmites* alone and *Melaleuca* alone (b) *Phragmites* plus *Melaleuca* without cutting of *Phragmites* shoots, (c) *Phragmites* plus *Melaleuca* subjected to one time *Phragmites* shoot cutting at 6 weeks during the 6-month experimental period, and (d) *Phragmites* plus *Melaleuca* with two times *Phragmites* shoot cuttings by a 6-week interval from commencement of the experiment until termination.

We transplanted the collected live rhizomes of *Phragmites* and *Melaleuca* seedlings into prepared experimental pots as soon as possible to avoid damage to the plant materials. The experimental pots were placed in a natural lit greenhouse, with a condition of 23 ± 3 °C and 12 ± 2 °C day/night temperature. We used an auto irrigation system to water the experimental pots and maintained soil moisture at 55 ± 5%, with a weekly soil moisture monitoring system. We randomized the experimental pots weekly by cleaning other seedlings except *Phragmites* and *Melaleuca*. We applied a destructive approach for biomass measurement and collected each individual of *Phragmites* and *Melaleuca* after 6 months of their growing for measurement of above-ground biomass (AGB) and below-ground biomass (BGB). Rhizome of *Phragmites* was collected for total non-structural carbohydrate (TNC) content to justify the role of carbohydrate for vegetative success of *Phragmites*. In addition to this, total nitrogen (TN) concentration in above-ground tissue of both species was measured to differentiate the nitrogen assimilation by both of the species.

### Laboratory incubation studies using field soil

Investigating AC-induced changes to soil nitrogen cycling rate and microbial activities [soil microbial biomass nitrogen (SMB-N) and dehydrogenase activity (DHA)] may be helpful explaining the changes in plant growth, as determined in the above experiment. The experiment was conducted in laboratory using nutrient enriched field soil and it was plant-free microcosms experiment to differentiate direct versus indirect effects of AC on the soil microbial functions. This was due to AC influencing soil effects on plant growth through allelochemicals absorption secreted by *Phragmites*^42,68^ and altering plant growth. We collected 15 soil cores from three high nutrient enriched *Phragmites* invaded populations in the Edithvale wetland (38°05′54.2′′S 145°08′24.8′′E) in December 2016. The wetland is located in south-eastern Melbourne which is listed as Ramsar site on August 2001. The Edithvale wetland is a mud-flat habitat, which is considered as high nutrient enriched soil and adversely impacted by urban development^39,73^. The main source of wetland nutrient enrichment is related to urban stormwater including industrial and agricultural runoff^74^. The soil was considered for this soil incubation experiment due to its high nutrient-enriched characteristics. The collected soil amended with and without AC was examined to compare how AC may change soil nitrogen cycling and microbial activities to understand the roles of nitrogen immobilization and microbial functions in controlling the competitiveness between *Phragmites* and *Melaleuca* in the above greenhouse studies.

The five invaded cores from each of the considered *Phragmites* population were collected and each individual core was kept in separate Ziploc bags. The samples were immediately transported to the laboratory with a laboratory ice box. Soils of the five replicates in each population were homogenised by sorting all debris and sieving (<2 mm). Before treatment, five subsamples of soil (10 g each) were collected from each composite and immediately extracted with 50 mL of 2.0 M KCl for the initial determination of extractable ammonium and nitrate. To half of the soil from each population, 70 g/L AC (the amount used for above greenhouse studies) was mixed into the soil. All soils with and without AC were rearranged in 5 replicates into the new plastic soil pot and maintained 50% soil moisture by tap water that was compatible with the field water-holding capacity. Then, the plastic soil pots were kept in dark at 22 °C for 30 days to simulate microbial activities and functions. After 30 days, the soils were removed, and subsamples were taken for specific laboratory analyses.

### Laboratory analysis

We used ion chromatograph to measure ammonium and nitrate concentrations in all soil extracts (Shimadzu Ion Chromotograph, Kyoto, Japan) following the method of Maynard *et al*.^75^. Net mineralization of N was calculated as extractable nitrate (NO_3_^−^) + ammonium in the incubated sample minus extractable nitrate + ammonium in the initial extracts. Net nitrification was calculated as extractable nitrate in the incubated sample minus extractable nitrate in the initial extracts. Microbial biomass nitrogen (MBN) and dehydrogenase activity (DHA) in treated soil were determined according to the procedures of Uddin & Robinson^71^. The alkaline persulfate oxidation method was used to determine the total nitrogen in above-ground plant tissue of the above greenhouse experimental samples of both species following the method proposed by Purcell & King^76^. We used phenol-sulphuric acid method for TNC measurement in *Phragmites* rhizome of the greenhouse experiment^77^.

### Statistical analyses

We applied analysis of variance (ANOVA) and nested ANOVA to assess the effects *Phragmites*, AC and *Phragmites* shoots cuttings on the measured variables of *Melaleuca*. In the case of *Melaleuca*, the model comprised of main effects of *Phragmites* & AC (effect of one of the independent variables on the dependent variables), and their interactions. In addition, the effects of cuttings of *Phragmites* shoots (nested within *Phragmites*), the cutting frequency of *Phragmites* shoots (nested within cutting), and their interactions with AC also formed the part of the model, which was analysed by the statistical methods of Uddin *et al*.^42^. In case of *Phragmites*, we used ANOVA models that included only cutting treatments of *Phragmites*, AC and their interactions. One-way ANOVA with LSD tests were used to compare the multiple means among treatments for different measured parameters of *Phragmites* and *Melaleuca*. We also used ANOVAs to examine the effects of AC addition on soil extractable nitrate and ammonium concentration, net mineralization and nitrification, microbial biomass-N, and DHA in the field soil incubation experiment. The model used included *Phragmites* populations and AC, and their interactions. Again, we used Real Statistics Resource Pack (Software plug-in, Microsoft Excel 2010) to investigate if the difference in slopes of regressions between AC treatments was statistically significant^78^. Furthermore, independent sample t-test was conducted to compare soil nitrogen indices within each population. We also applied Levene’s test of equality to check the homogeneity of variance of the used data (square root transformed if necessary) for both of *Phragmites* and *Melaleuca*. We considered *P* ≤ 0.05 as significant for all experimental data analyses by using statistical software IBM SPSS statistics 24.0.

## Results

### Plant responses to activated carbon and shoot cuttings of *Phragmites*

**Figure 1:**
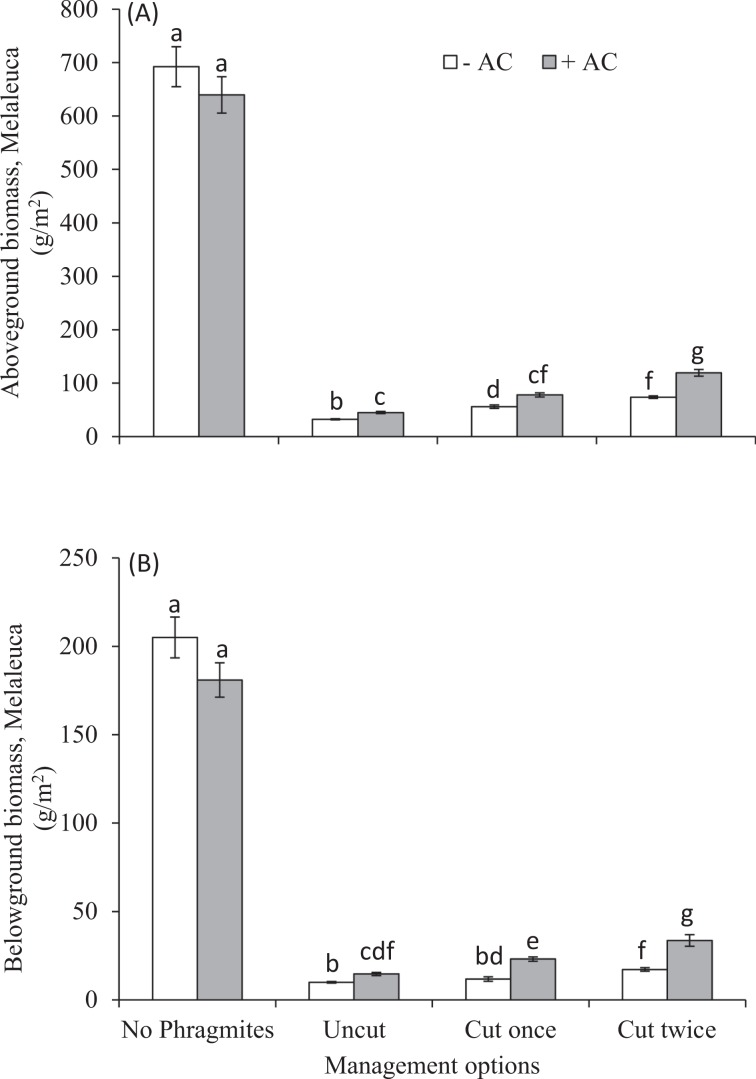
Effects of management strategies of *Phragmites* (cuttings of *Phragmites* shoots) either in AC-untreated or AC-treated on (**A**) above-ground biomass (g/m^2^), and (**B)** below-ground biomass(g/m^2^) of *Melaleuca* growing single or mixed with *Phragmites* in experimental pots. Each bar is the mean ± *SE* (*n* = *5*). Different letters in each bar indicate significantly differences (*P* ≤ 0.05).

**Figure 2:**
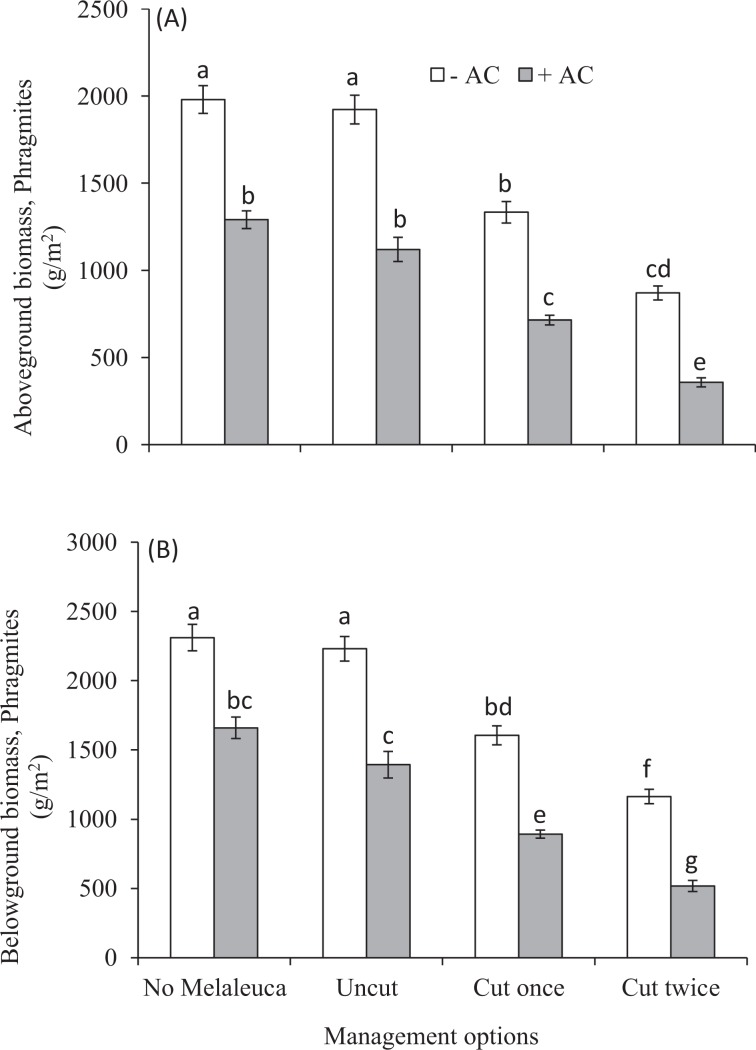
Effects of management strategies of *Phragmites* (cuttings of *Phragmites* shoots) either in AC-untreated or AC-treated on (**A)** Above-ground biomass (g/m^2^), and (**B)** below-ground biomass (g/m^2^) of *Phragmites* growing single, or mixed with *Melaleuca* in the experimental pots. Each bar is the mean ± *SE* (*n* = *5*). Different letters in each bar indicate significantly differences (*P* ≤ 0.05).

The aboveground and belowground biomass of *Melaleuca* was negatively affected significantly by competition with *Phragmites* (Table 1, Fig. 1A,B). The average *Melaleuca* AGB decreased from 692.24 ± 37.38 to 32.29 ± 1.03 g/m^2^ and 6399.55 ± 34.09 to 45.02 ± 2.06 g/m^2^ between mono (only *Melaleuca*) and mixed uncut cultures (*Phragmites* and *Melaleuca* grown together), either AC-untreated or AC-treated, respectively (Fig. 1A,B). In case of BGB, it reduced from 205.02 ± 16.55 to 9.92 ± 0.44 g/m^2^ and 180.94 ± 9.74 to 14.61 ± 0.90 g/m^2^ respectively (Fig. 1A,B). AC had no significant growth effect on *Melaleuca* in mono-culture whereas it had significant effect on mixed uncut cultures (Fig. 1A,B). Again, AC had significant negative growth effect on *Phragmites* in both mono and mixed uncut cultures (Table 2, Fig. 2A,B). The AGB and BGB of *Phragmites* was reduced from 1979.88 ± 79.81 to 1290.37 ± 50.95 g/m^2^ and 2311.22 ± 95.40 to 1660.33 ± 77.55 g/m^2^ between mono and mixed uncut cultures without and with AC, respectively. The addition of AC had different outcomes on the competitive interactions between *Phragmites* and *Melaleuca* biomass, but it was noticed that *Melaleuca* growth increased due to AC in uncut regime (Figs. 1 and 2). The interactive effects of AC and *Phragmites* were significant effect on biomass of *Melaleuca* (Table 1).

**Table 1:** Results of ANOVA and nested ANOVA analysing treatments of *Phragmites*, activated carbon, and management strategies of *Phragmites* (cutting of *Phragmites* shoots) on dry weight of aboveground biomass (AGB), belowground biomass (BGB), total biomass (TB), and total nitrogen (TN) concentration of AGB of *Melaleuca*.

Source^a^	*df*1, *df*2	AGB		BGB		Total biomass		TN concentration	
		*F*	*P*	*F*	*P*	*F*	*P*	*F*	*P*
*Phragmites*	1, 36	583.23	<0.001	757.70	<0.001	631.39	<0.001	496.64	<0.001
Activated carbon	1, 36	0.83	0.36	2.27	0.14	1.07	0.31	60.14	<0.001
P × AC	1, 36	3.95	≤0.05	11.07	<0.05	5.19	<0.05	19.67	<0.001
Cutting (P)	1, 37	47.20	<0.001	12.41	<0.001	36.48	<0.001	27.25	<0.001
C frequency (C, P)	1, 36	13.58	<0.001	6.66	<0.05	11.56	<0.05	6.36	<0.05
C (P) × AC	2, 34	6.71	<0.05	11.56	<0.001	8.01	<0.001	1.12	0.33
C frequency (C, P) × AC	3, 32	9.95	<0.001	11.61	<0.001	10.72	<0.001	1.96	0.13

^a^AC = activated carbon, C = cutting, P = *Phragmites*.

**Table 2:** Results of ANOVA and nested ANOVA analysing treatments of activated carbon and management strategies of *Phragmites* (cutting of *Phragmites* shoots) on dry weight of aboveground biomass (AGB), belowground biomass (BGB), total biomass, total nitrogen (TN) concentration of AGB and total non-structural carbohydrate (TNC) of rhizome in *Phragmites* growing in experimental communities with *Melaleuca*.

Source^a^	*df*1, *df*2	AGB		BGB		Total biomass		TN concentration		TNC	
		*F*	*P*	*F*	*P*	*F*	*P*	*F*	*P*	*F*	*P*
Activated carbon	1, 34	102.59	<0.001	94.70	<0.001	118.66	<0.001	136.18	<0.001	34.24	<0.001
Cutting	2, 34	70.62	<0.001	75.01	<0.001	87.85	<0.001	47.27	<0.001	145.34	<0.001
AC * C	2, 34	1.18	0.32	0.54	0.58	0.87	0.43	0.41	0.66	2.47	0.09
C frequency (C)	1, 36	6.10	<0.05	5.01	<0.05	5.81	<0.05	6.33	<0.05	15.83	<0.001
C frequency (C) * AC	3, 32	2.17	0.11	0.74	0.53	1.84	0.16	1.40	0.26	7.16	<0.001

^a^AC = activated carbon, C = cutting.

The cutting had significant negative effect on biomass of *Phragmites*, and consequently, it increased biomass of *Melaleuca* (Tables 1 and 2, and Figs. 1 and 2). The single *Phragmites* shoot cuttings reduced 29% (4152.37 ± 100.48 to 2938.37 ± 129.03 g/m^2^) & 36% (2512.69 ± 158.87 to 1607.14 ± 50.44 g/m^2^) total biomass of *Phragmites* and increased 60% (42.20 ± 1.25 to 67.71 ± 4.34 g/m^2^) & 69% (59.63 ± 2.90 to 100.90 ± 5.02 g/m^2^) total biomass of *Melaleuca* in without and with AC, respectively (Figs. 1 and 2). Again, double shoot cuttings of *Phragmites* reduced 51% & 65% total biomass of *Phragmites* and increased 115% & 157% total biomass of *Melaleuca* in without and with AC, respectively (Figs. 1 and 2). The same cutting had also significant effects on biomass of *Phragmites* and *Melaleuca* in AC-untreated and AC-treated experimental pots (Figs. 1 and 2) (*P* ≤ 0.05).

**Figure 3:**
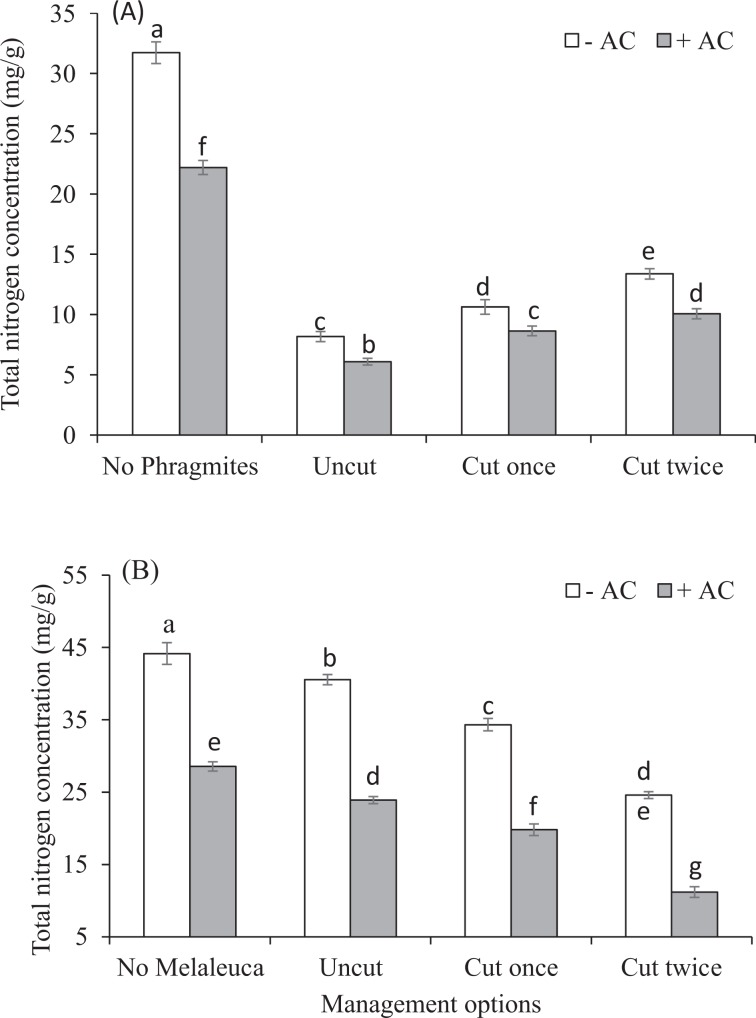
Effects of management strategies of *Phragmites* (cuttings of *Phragmites* shoots) either in AC-untreated or AC-treated on Total nitrogen (TN) concentration (mg/g) of (**A**) *Melaleuca* and (**B**) *Phragmites* in above-ground biomass growing single, or mixed with *Melaleuca* in the experimental pots. Each bar is the mean ± *SE* (*n* = *5*). Different letters in each bar indicate significantly differences (*P* ≤ 0.05).

Total nitrogen concentration in above-ground biomass of *Phragmites* was greater than *Melaleuca* (Fig. 3A,B). TN concentration of *Melaleuca* was negatively affected significantly with *Phragmites* competition and AC treatments (Table 1). Cutting and cutting frequency of *Phragmites* shoots also significantly increased the TN concentration of *Melaleuca* among mixed cultures, whereas the TN concentration of *Phragmites* was decreased with both AC-untreated and AC-treated cultures (Fig. 3A,B), but however, no significant interactions between AC and cutting regimes were found (Table 1). The average *Melaleuca* TN concentration was reduced from 31.72 ± 0.90 to 8.17 ± 0.42 mg/g with competition of *Phragmites* without AC, which was 74%, whereas with AC it was 72% (22.20 ± 0.58 to 6.09 ± 0.28 mg/g). Again, cutting of the *Phragmites* shoots has significant positive effect in increasing the TN concentration of *Melaleuca*, which was an average from 8.17 ± 0.42 to 13.77 ± 0.43 mg/g without AC and 6.09 ± 0.28 to 10.06 ± 0.42 mg/g with AC, whereas TN concentration of *Phragmites* was reduced from 40.55 ± 0.71 to 24.59 ± 0.48 mg/g without AC and 23.90 ± 0.49 to 11.19 ± 0.74 mg/g with AC.

**Figure 4:**
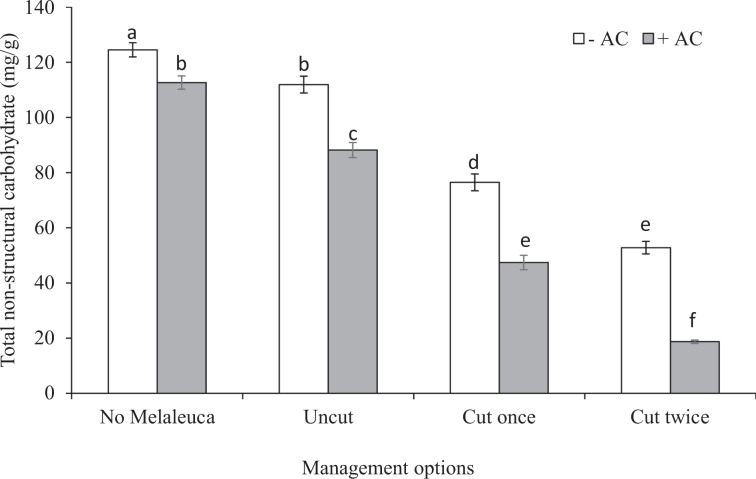
Effects of management strategies of *Phragmites* (cuttings of *Phragmites* shoots) either in AC-untreated or AC-treated on total non-structural carbohydrate (TNC) (mg/g) of *Phragmites* rhizome growing single, or mixed with *Melaleuca* in the experimental pots. Each bar is the mean ± *SE* (*n* = *5*). Different letters in each bar indicate significantly differences (*P* ≤ 0.05).

The concentrations of TNC in *Phragmites* rhizomes strongly differed between the treatments (Table 2, Fig. 4). The imposed management regimes affected the levels of TNC, and TNC concentrations were significantly higher at without AC than with AC. Single shoot cuttings of *Phragmites* reduced 32% (111.88 ± 3.05 to 76.46 ± 3.03 mg/g) & 46% (88.19 ± 2.75 to 47.41 ± 2.62 mg/g) TNC concentration in without and with AC respectively, whereas they were 53% & 79% for double shoot cuttings (Fig. 4). However, cutting of *Phragmites* shoot had significant negative effects on TNC at both levels of AC. There were no competition effects on TNC at both AC levels (Table 2), but significant interactions between AC and cutting management were found for TNC indicates that the effects of these management regimes were effective (Table 2).

### Relationships among measured variables

**Figure 5:**
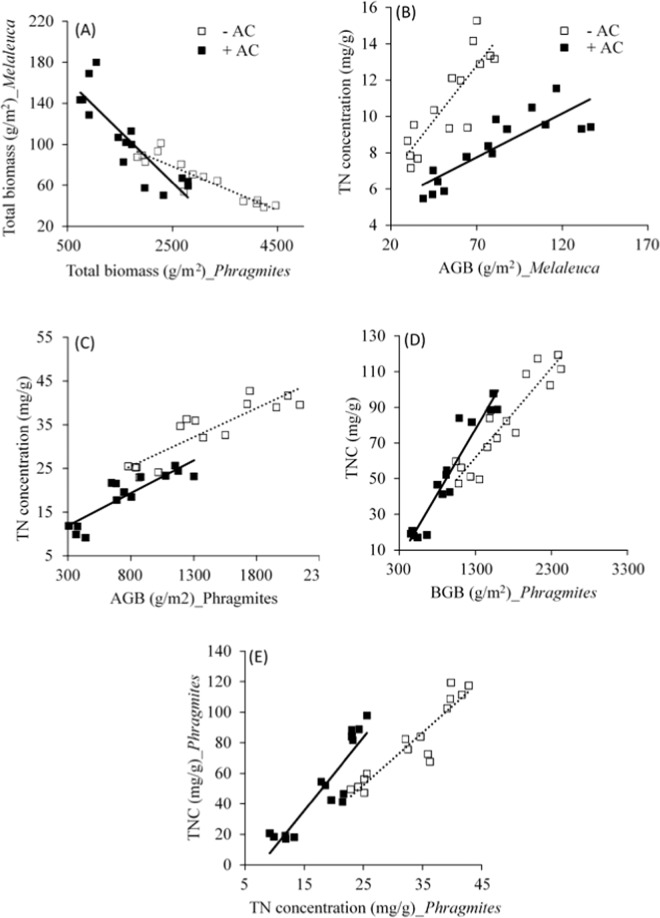
Regression analyses between (**A**) total biomass of *Phragmites* and *Melaleuca*; (**B**) above-ground biomass (AGB) of *Melaleuca* and total nitrogen (TN) concentration of AGB *Melaleuca*; (**C**) above-ground biomass (AGB) of *Phragmites* and total nitrogen (TN) concentration of AGB *Phragmites*; (**D**) below-ground biomass (BGB) of *Phragmites* and total non-structural carbohydrate (TNC) of *Phragmites* rhizome; and (**E**) total nitrogen (TN) concentration of AGB *Phragmites* and total non-structural carbohydrate (TNC) of *Phragmites* rhizome grown in mixed (*Phragmites* and *Melaleuca* together) cultures whether *Phragmites* subjected to different management strategies (cuttings of *Phragmites* shoots) either in AC-untreated or AC-treated experimental pots.

There was significant negative correlation between *Phragmites* and *Melaleuca* total plant biomass in all experimental pots (Fig. 5A). It was observed that *Phragmites* acquired greater biomass in AC-untreated pots compared to AC-treated pots across the cutting frequency (Fig. 5A). In contrast, *Melaleuca* showed the opposite scenario regarding biomass accumulation. The significant difference was found between slopes of the regression lines of AC-untreated and AC-treated pots (*t = *3.47) with *df* 26 and *α* 0.05, and the stronger negative correlation was noticed in AC-untreated pots (F = 57.12, *df* = 1,12, *p* > 0.001; *r*^2^ = 0.85) compared to AC-treated pots (F = 36.55, *df* = 1,12, *p* > 0.001; *r*^2^ = 0.76) (Fig. 5A). Again, a significant positive relationship between aboveground biomass and total nitrogen concentration of *Melaleuca* and *Phragmites* in mixed culture both in AC-untreated and AC-treated was found, but the responses were different (Fig. 5B,C). The significant difference was found between slopes of the regression lines of AC-untreated and AC-treated pots for *Melaleuca* (*t* = 3.38) (Fig. 5B), but not for *Phragmites* (*t = *0.5) with *df* 26 and *α* 0.05 (Fig. 5C). The most positive relationships were found for *Phragmites* (AC-untreated: F = 45.19, *df* = 1,12, *p* > 0.001, *r*^2^ = 0.79; AC-treated: F = 39.44, *df* = 1,12, *p* > 0.001; *r*^2^ = 0.76) compared to *Melaleuca* (AC-untreated: F = 31.24, *df* = 1,12, *p* > 0.001, *r*^2^ = 0.72; AC-treated: F = 27.75, *df* = 1,12, *p* > 0.001; *r*^2^ = 0.69) both in AC-untreated and AC-treated mixed cultures (Fig. 5B,C). Furthermore, it was found that there was a significant positive correlation between below-ground biomass of *Phragmites* and total non-structural carbohydrates in both AC-untreated and AC-treated mixed communities (Fig. 5D). The slope difference between AC-untreated and AC-treated was significant (*t = *2.69) with *df* 26 and *α* 0.05 that indicates AC had a significant effect in influencing the carbohydrate storage in rhizomes (Fig. 5D). The stronger positive relation was noted in AC-treated pots (F = 131.17, *df* = 1,12, *p* > 0.001; *r*^2^ = 0.91) compared to AC-untreated pots (F = 75.77, *df* = 1,12, *p* > 0.001; *r*^2^ = 0.86) (Fig. 5D). Again, the strong positive relationship between TN concentration of AGB and TNC level of BGB in *Phragmites* was noticed in AC-untreated (F = 67.36, *df* = 1,12, *p* > 0.001; *r*^2^ = 0.85) compared to AC-treated (F = 49.97, *df* = 1,12, *p* > 0.001; *r*^2^ = 0.82) mixed communities (Fig. 5E), but the slope difference was not significant (*t* = 1.85) with *df* 26 and *α* 0.05.

### Soil responses to activated carbon

**Figure 6:**
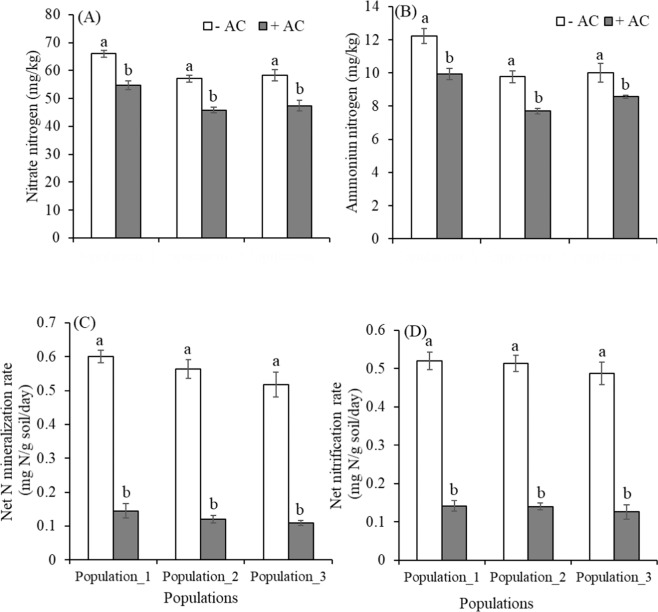
Extractable (**A)** nitrate and (**B)** ammonium concentrations, (**C)** net nitrogen (N) mineralization rate, and (**D)** net nitrification rate in activated carbon (AC) unamended and amended pots of different populations. Each bar is the mean ± *SE* (*n* = *5*). Different letters in each bar indicate significantly differences (*P* ≤ 0.05).

**Figure 7:**
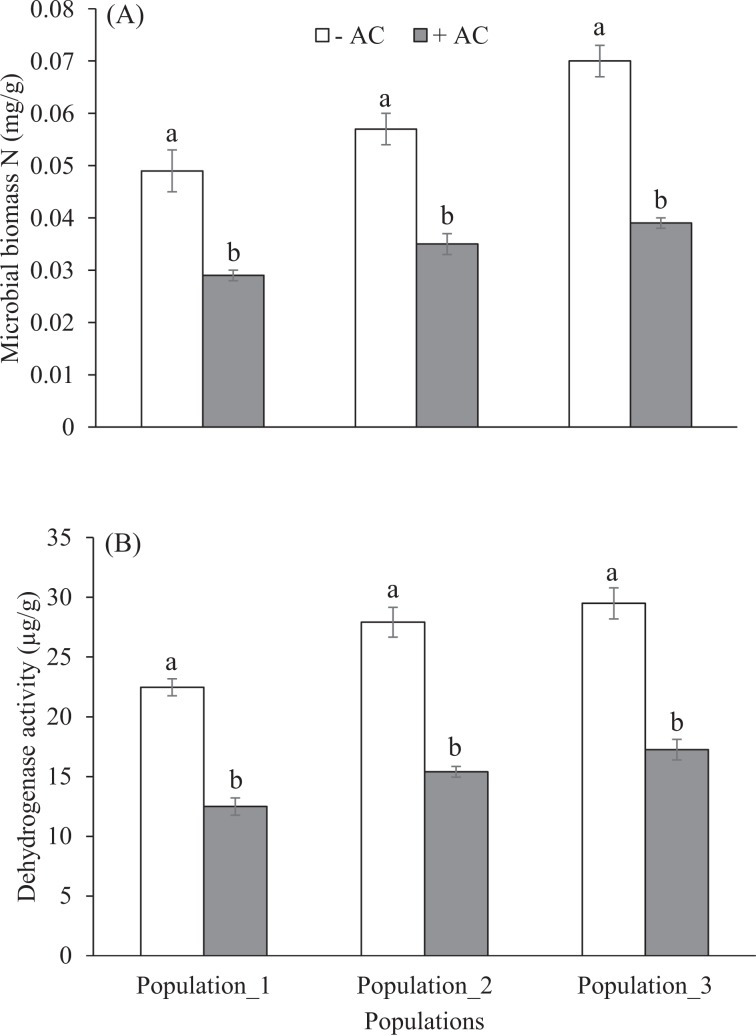
Soil (**A)** microbial biomass nitrogen (SMB-N) and (**B)** dehydrogenase activity in activated carbon (AC) unamended and amended pots of different populations. Each bar is the mean ± *SE* (*n* = *5*). Different letters in each bar indicate significantly differences (*P* ≤ 0.05).

Concentrations of extractable NO_3_-N and NH_4_-N were greatly reduced in pots treated with AC relative to unamended pots at the end of the experiment in all populations (Table 3, Fig. 6A,B). Soils amended with AC reduced an average 19% and 17% NO_3_−N and NH_4_-N respectively. The reductions of NO_3_-N were from 66.07 ± 1.03 to 54.72 ± 1.59 mg/kg, 57.09 ± 1.21 to 45.89 ± 1.00 mg/kg and 58.28 ± 1.97 to 47.45 ± 1.82 mg/kg in population_1, population_2 and population_3 respectively (Fig. 6A). The NH_4_-N was reduced by an order of scale, from 12.24 ± 0.45 to 9.94 ± 0.34 mg/kg, 9.77 ± 0.36 to 7.70 ± 0.17 mg/kg, 10.01 ± 0.55 to 8.57 ± 0.10 mg/kg in population_1, population_2 and population_3 respectively (Fig. 6B). AC addition decreased both net N mineralization and net N nitrification significantly, which was on an average 77% and 73% respectively compared to without AC pots in all populations (Table 3, Fig. 6C,D), but *Phragmites* population reduced significantly only net N mineralization (Table 3). Microbial biomass N was significantly higher in AC unamended pots than amended pots, which were 40.81, 38.59 & 44.28% increment in population_1, population_2 and population_3, respectively (Table 3, Fig. 7A). However, the microbial biomass N was significantly influenced by *Phragmites* populations, but there is no significant interaction between AC and populations (Table 3). The microbial biomass N decreased on an average 41% at AC-amended soil compared to AC unamended soil. Dehydrogenase activity was also significantly lower in the AC-amended soil by an average 43.57% than in the AC-unamended soil (Fig. 7B). AC-amendment decreased dehydrogenase activity most significantly in population_2 among three populations (Fig. 7B). The overall reduction of dehydrogenase activity indicated a decrease in the microbial metabolism in the soil due to less mineralization and nitrification rate in the AC-amended soil (Table 3, Fig. 6C,D). All parameters except net nitrification in soil significantly varied across the AC-amendment and populations but no interactive effects (AC × *Phragmites* populations) were observed (Table 3).

**Table 3:** Results of ANOVA analysing treatments of activated carbon, *Phragmites* population, and their interactions on soil nitrate (NO_3_^−^N), ammonium (NH_4_^+^-N), microbial biomass nitrogen (MB-N), and dehydrogenase activity (DHA) in nutrient enriched *Phragmites* invaded soil.

Source^a^	*df*1, *df*2	NO_3_^−^N		NH_4_^+^-N		Net mineralization		Net nitrification		MB-N		DHA	
		*F*	*P*	*F*	*P*	*F*	*P*	*F*	*P*	*F*	*P*	*F*	*P*
Activated carbon	1, 24	81.52	<0.001	43.09	<0.001	601.20	<0.001	478.51	<0.001	163.49	<0.001	231.01	<0.001
*Phragmites* population	2, 24	20.18	<0.001	23.34	<0.001	3.44	<0.05	0.80	0.46	20.96	<0.001	21.07	<0.001
AC * PP	2, 24	0.016	0.98	0.76	0.47	0.08	0.92	0.09	0.91	1.20	0.32	1.11	0.35

^a^AC = activated carbon, PP = *Phragmites* population.

## Discussion

### Plant responses

In nutrient-enriched wetlands, *Phragmites* seedlings and adult plants grow larger and produce more stems, florets, inflorescences and thus, seeds with rhizome, further benefiting the invasion^79^. We hypothesized that reducing nitrogen availability in nutrient-rich soil via AC addition followed by *Phragmites* shoot cuttings may reduce *Phragmites* growth, and thereby, may favour growth of associate plant species *Melaleuca*. So, controlling growth of *Phragmites* in nutrient-rich wetlands, our experimental results demonstrate that AC-amended soil followed by *Phragmites* shoot cuttings increased competitiveness of *Melaleuca* over *Phragmites* growth (Fig. 1). On the contrary, *Phragmites* competitively overwhelmed *Melaleuca* growth in AC unamended soil (Fig. 1), which is aligned with other studies in prairie restoration^80^. The AC additions in our studies reduced biomass of *Phragmites* that indicates the reduction of invasibility of the plant community invaded by *Phragmites*. Conversely, AC increased biomass of *Melaleuca* in mixed culture, which suggest that AC had negative impact on *Phragmites* allowing *Melaleuca* to be more competitive.

Our results are largely consistent with the fluctuating resource hypothesis of invasibility proposed by Davis *et al*.^81^, whom suggests that a plant community is more susceptible to invasion when there is an increase in the amount of unused resources. This condition also increases the relative competitive ability of invasive species in a plant community^82^. In our experimental results, the increase of *Melaleuca* biomass in AC-amended mixed cultures indicated a relatively low positive outcome because *Melaleuca* is a tree with lengthy lifespan, it requires longer time to achieve more biomass than *Phragmites*, a perennial grass. *Melaleuca* had also better growth (shown in single growth experiment) suggesting that with the correct growth condition, it would successfully establish in its community. As a result, it can be said *Melaleuca* may establish and grow properly in *Phragmites*-dominated wetland ecosystems with AC-amendment followed by reducing biomass of *Phragmites*.

The success of the establishment of plants depends on mechanisms, and the mechanisms are species-specific and contextual^83^. It is difficult to establish a single management approach to an ecological restoration initiative, requiring integrated control measures to restore the degraded ecosystem. This would be practical if the targeted plant, for example, *Phragmites* has persistent communities^84^. In our studies, however, only the AC addition reduced *Phragmites* biomass and increased *Melaleuca* biomass, but AC with cutting of *Phragmites* shoots enhanced the magnitude of reduction for *Phragmites* biomass. Subsequently, this approach increased the biomass of *Melaleuca* more significantly suggesting combined management (AC with cuttings of shoots) was more effective than only AC addition to reduce the invasibility of the community by *Phragmites*. *Melaleuca* experienced comparatively less impact by *Phragmites* in AC-amended pots compared with AC-unamended cutting treatments pots (Fig. 1). It may imply that AC-induced low N availability in soil had less effect on the competitive ability of *Melaleuca* and *Phragmites*. It is assumed that *Melaleuca* achieved benefits to establishment and growth in cutting experiments with AC-amendment through nitrogen immobilization, which was also effective in reducing *Phragmites* competitive ability. Thus, our results were compatible with findings of Perry *et al*.^85^ whom found lowering N availability through carbon amendment reduced the competitive ability of invasive species, *Phalaris arundinacea*. As a result, we can assume that the effects of AC addition with repetitive cutting of *Phragmites* shoot on *Melaleuca* growth is explained through N immobilization.

The increasing trend of total N concentration in AGB of *Melaleuca* and decreasing level in *Phragmites* AGB among treatments indicates that AC associated with cuttings of *Phragmites* shoots offered competitive ability of *Melaleuca* by up-taking more available nitrogen over *Phragmites*. This is aligned with other studies, that found invasive species attained comparatively less nitrogen content than native in the used treatments^85,86^. We also examined the effects of treatments on carbohydrates reserves in *Phragmites* rhizome that allow *Phragmites* to re-sprout in the next spring growth. The treatments reduced significantly *Phragmites* biomass (above and belowground) and carbohydrate reserves in rhizome. Additionally, our laboratory incubation experiment showed that AC also had direct impact on soil N availability. This assumes that it might be a basic function of both N-availability in soil and carbohydrate reserve in rhizome for competitive advantage of *Phragmites* in the plant community. This implies *Phragmites* establishment and growth might be restricted by carbohydrate reserve in rhizome and N-availability in soil. This finding is aligned with the studies of Kleijn *et al*.^87^, whom found available soil N supplemented by stored carbohydrate reserves was responsible for vegetative success of *Veratrum album*.

The negative correlation between *Phragmites* and *Melaleuca* total biomass (Fig. 5A), and the more reduction of *Melaleuca* biomass in AC-untreated compared to AC-treated experimental pots (Fig. 1A,B) indicates that AC with cutting of *Phragmites* shoots may promote the productivity of *Melaleuca*, which is aligned other studies^62^. The studies found carbon amendment reduced N availability in soil, and thereby, reduced competition between native and exotic plant, and provided benefit to the native. The more positive relationship between nitrogen concentration and above-ground biomass in *Phragmites* (Fig. 5B,C) indicates responses of *Phragmites* to N availability was greater than *Melaleuca*, which may provide it indirect benefit. *Melaleuca* however, may experience less negative effects than *Phragmites* for the reduction of N availability, which is supported by the studies of Alpert and Maron^88^ and Alpert^64^. They found carbon addition may reduce ecosystem invasiblity by invasive grass through lowering nitrogen availability and promoting native plants growth for ecosystem restoration.

Furthermore, AC treatment with cuttings influence TNC levels of rhizomes indicates that the management options may work in combination to slowly reduce carbohydrate reserves of *Phragmites* rhizome, thus ultimately negatively influencing the sprouting for the next growth. The results are aligned with the studies of Moyo *et al*.^89^, whom found cutting management had significant negative effect on TNC level. The reducing level was amplified with cutting frequency that ultimately affected shoot production. Additionally, the positive relationships among rhizome biomass, TNC of *Phragmites* rhizome and TN concentration of *Phragmites* AGB (Fig. 5D,E) indicate that reducing rhizome biomass through cutting and AC-amendment may act as a negative driver to re-sprout for *Phragmites*, which ultimately provides benefits to *Melaleuca*. The findings are similar with other studies of Druege *et al*.^90^ whom found nitrogen concentration and TNC level are positively correlated, and cutting frequency may reduce TNC level in *Chrysanthemum* plant species.

### Soil responses

In our study, extractable inorganic N concentrations (nitrate and ammonium concentrations) were lower in AC pots relative to unamended pots, consistent with the studies of Blumenthal *et al*.^59^ and Morghan *et al*.^91^, where addition of AC as sucrose and/ or sawdust has shown reduced soil inorganic N levels, though the efficacy depends on initial soil fertility, quantity and form of AC added. Again, our results showed that AC additions stimulated microbial N-immobilization by reducing rates of net N mineralization and net nitrification, which is consistent with the findings of Torok *et al*.^92^, who found nitrogen immobilization by carbon acts to stimulate the restoration process in a grassland community. The results of our laboratory incubation are compatible to explain the underlying mechanism of nitrogen cycling in the first plant community experiment as the N level in the used field soil for incubation experiment was high^39^.

The MBN significantly decreased in the AC-amended soil in all populations in our study, which is supported by other studies^93,94^. For example, Zhang *et al*.^93^ found the MBN decreased with AC addition at a rate of 4.5 t/ha/yr in the field at most sampling dates and soil depths compared to the unamended soil treatment, whereas Zavalloni *et al*.^95^ and Dempster *et al*.^96^ found AC amendment had no significant impact on MBN. Again, AC increased soil MBC in our studies supported by Ma *et al*.^97^ and Gebhardt *et al*.^98^ whom found addition of carbon as a soil amendment significantly increased MBC and thereby, altered plant biomass. Therefore, the increased MBC and decreased MBN indicates that AC in soil acted as a carbon source rather than a nitrogen source for microbes in soil substrates. As a result, this had a significant influence on N cycling, for example, increasing microbial N immobilization, which is also aligned with our results. AC is not only responsible for reducing soil N level, but it may work in soil through different mechanisms. For example, some studies assumed that AC changes plant growth by sequestering allelochemicals, that was found in previous *Phragmites* studies including ours^42^, and altering microbial functions through changes of microbial communities^99,100^. In this case, soil DHA may be used as an indicator of overall soil microbial activity^101^. In our study, AC caused direct changes to soil microbial functions, which were observed in our plant-free microcosms laboratory incubation experiment. Our studies showed AC decreased DHA compared to unamended soil for each population, which might be linked to decreasing the microbial symbionts associated with *Phragmites* success, which is explained by *Centaurea* species to support this argument^102,103^.

### Mechanisms involved in reducing invasibility

It is common for soil-N availability to influence positively for rapid shoot growth in plant and ultimately, increasing plant biomass^39,87^. In AC-amended soil, *Melaleuca* attained relatively higher biomass and N level with increasing cutting frequency of *Phragmites* shoots, whereas *Phragmites* experienced a lower level. This implies N-poor soil might be a good condition for the competitive ability of *Melaleuca*. It also indicates that N-use efficiency of *Melaleuca* in AC-amended soil would be higher than *Phragmites*, which was also supplemented by the *Phragmites* shoot cuttings. The findings of our studies are compatible with Perry *et al*.^85^, whom found native plant *Carex hystericina* achieved greater N-uptake and biomass compared to invasive species *Phalaris arundinacea* in carbon-treated soil. Again, Chapin *et al*.^104^ found both nutrient level in soil and carbohydrate reserves play an important role to establishment of the plant, but the rapid growth is regulated completely by carbohydrate reserves. In this case, our results support the idea that reduced carbohydrate and nitrogen level in AC-amendment followed by *Phragmites* shoot cuttings influenced negatively the competitive ability and plant growth of *Phragmites*, whereas it allowed *Melaleuca* the opportunity to grow and establish.

Furthermore, it is assumed that N availability in soil and carbohydrate reserve in rhizome are responsible in facilitating *Phragmites* invasion and to completing its growing season in the short term. In our study, it appeared that while shoot cutting with AC treatments significantly reduced carbohydrates simultaneously of *Phragmites*, the remaining carbohydrate reserves may act as a survivor for the next spring growth. This was noted in another study related to perennial *Veratrum album* by Schaffner *et al*.^105^. So, it is important to continue the process until next growing season of *Phragmites*, but it is subject to context to making the approach effective in control of *Phragmites* in nutrient enriched condition.

### Implications for *Phragmites* control

*Melaleuca* growth improvement over *Phragmites* in AC-amended soil followed by cutting treatments suggests that this condition lowered soil N availability through N-immobilization that reduced *Phragmites* growth. This situation is shown between *Phragmites* and *Melaleuca* interactions, and requires further research to demonstrate the interactions between other associated plant species and *Phragmites* in wetlands within this condition. Creating N-immobilization in *Phragmites* dominated wetland restoration technique needs active reduction of soil N availability by using AC. Additionally, N availability in wetlands may be reduced by frequent cutting of *Phragmites* shoots before starting a restoration initiative, which supports the studies of Verhoeven & Schmitz^106^, whom found frequent mowing was effective in lowering the nutrient condition, that controls the plant growth^8^. Nitrogen immobilization technique via activated carbon has potential to reduce invasibility and provide positive effect on native plant growth in ecological restoration initiatives^107,108^. The challenge is however, that this restoration technique needs higher amount of AC for larger scale implications, thus entailing higher cost. Maintaining the reduced N-availability in soil presents additional challenge, as many environmental processes (mineralization) and factors (surface runoff due to agriculture & industrialization) actively work to increase the N-inputs in wetlands. For this reason, it is essential to develop a new technique to reduce nitrogen level in *Phragmites* dominated wetland, which is partially supplemented by frequent shoot cutting of *Phragmites*. The minimum level of AC required for lowering N level in wetland soil prior to restoration initiative also needs to be determined, though it is contextual. Furthermore, it is essential to prevent the N-source to *Phragmites* dominated wetlands, which can be manipulated through vegetation buffering and landscaping^109^. Further study is recommended to investigate the wide range of N-assimilation between *Melaleuca* and *Phragmites* under this condition to find out the difference amongst them.

## Conclusion

Our past studies^39^ showed *Phragmites* is more dominating in nutrient-rich compared to nutrient-poor condition which provides further evidence in supporting the hypothesis that nutrient enrichment facilitates invasion of  *Phragmites*. Based on this scenario, this study demonstrated whether nutrient (nitrogen) manipulation may provide a competitive advantage to *Melaleuca* over *Phragmites* that might be an operative and effective restoration tool to restore the *Phragmites*-dominated wetlands. Our results suggest that AC addition in soil followed by frequent *Phragmites* shoot cuttings may provide competitive advantage for *Melaleuca* over *Phragmites*. Additionally, it is  assumed that soil nitrogen immobilization due to AC addition may be responsible for *Phragmites* growth reduction leading to facilitating *Melaleuca* growth. However,  soil inorganic nitrogen availability was not measured in the plant microcosms experiment, but it was addressed by plant-free microcosms and supported by the studies of Perry *et al*.^85^ whom found inorganic nitrogen was reduced in carbon-enriched soil by 10 to 30 mg/kg in a competitive experiment between invasive *Phalaris arundinacea* and a native sedge *Carex hystericina*. Furthermore, Torok *et al*.^92,110^ found immobilization of soil nitrogen by carbon addition was effective for the sandy grassland restoration. To the best of our knowledge, these types of experimental studies regarding *Phragmites* management are the first where nutrient (nitrogen) manipulation through AC followed by *Phragmites* shoot cuttings have demonstrated the examination of competitive ability between *Phragmites* and associated native species, further explained by microbial interactions that may mediate the growth of native over dominating species *Phragmites*.

## Supplementary information

Dataset 1.

## Data Availability

Data will be available where applicable.
