# Supplementary material for: Nitrogen immobilization may reduce invasibility of nutrient enriched plant community invaded by Phragmites australis
Source: Sci Rep. 2020 Jan 31;10:1601. doi: 10.1038/s41598-020-58523-4 (PMC6994487; doi:10.1038/s41598-020-58523-4)
Supplement: Supplementary file 1 — Dataset 1. [file 41598_2020_58523_MOESM1_ESM.pdf]

SREP-18-48613: Nitrogen immobilization may reduce invasibility of nutrient enriched plant community invaded by *Phragmites australis*

Md. Nazim Uddin[1, 2*], Randall William Robinson[2], and Takashi Asaeda[1]

Appendix S1. The condition of "earth-wise growing essentials potting mix" used in the experiments.

Potting mix components	Value (unit)
Composted pine bark	5 (%)
Earthwise compost*	95 (%)
Ferrous sulphate	1 (kg/m^3)
Gypsum	0.6 (kg/m^3)

*Earth-wise compost is made from sawdust, cane dust and mill-mud. Mill-mud is a residue from the processing of sugar cane.

Typical analysis of potting-mix soil used in the experiments

Potting mix properties	Value (unit)
Air filled porosity.	18 (%)
Water holding capacity	56 (%)
pH	5.8-6.2
EC	1.6-2.0 (dS/m)
Nitrogen drawdown index	> 0.5
Toxicity	> 0.7 (%)
Nitrogen	10 (mg/L)
Phosphate	2.7 (mg/L)
Potassium	30 (mg/L)
Calcium	150 (mg/L)
Magnesium	55 (mg/L)
Iron	25 (mg/L)

Source: Australian Prime Fibre Pty. Ltd, 186 Glenmount Road, Tanawah, QLD- 4556, Australia
